# 

**Published:** 1871-04

**Authors:** 


### BOOK NOTICES.

"Life at Threescore and Ten" is a beautiful 40 cent volume of the AMERICAN TRACT SOCIETY, 150 Nassau Street, New York, by the late Dr. BARNES, of Philadelphia. It is a mine of reflection and of devotional feeling to every Christian heart.

THE NATIONAL TEMPERANCE SOCIETY and Publication House, 172 William Street, New York, J. N. Stearnes, agent, have issued "Frank Spencer's Rule of Life, and how it led to his Prosperity." Fact, not fiction. 179 pp. 35 cts.

From the teeming press of HURD & HOUGHTON comes "From Fourteen to Fourscore," by Mrs. S. W. JEWETT, 416 pp.; and any intelligent reader "from fourteen to fourscore" can read this deeply interesting and suggestive book with profit to head and heart about life's experiences. Also, "Stories and Tales," by HANS CHRISTIAN ANDERSEN, 532 pp., with many interesting illustrations. The same author's "Wonder Stories for Children," had a wide sale and a very general public appreciation. Both books are notable for their purity, their high standard of morals, and for vividness and accuracy of delineation, whether of character, history, or incident. Safe, useful, and instructive books for all families.

"Sam Shirk: A Tale of the Woods of Maine," by GEORGE H. DEVEREUX. $1.75.

"The Southern Presbyterian Review," Columbia, S. C., No. 1, vol. 22, is edited with ability and discrimination, grappling with the truths of the times with vigor and judgment. "The Dealings of Christ with the Chinese Nation," by the Rev. M. H. HOUSTON, Hanchau, China. "Manses, or Minister's Houses." "The Conditions, Wants, and Prospects of the Church," etc., $5 a year.

The last number of the "London Lancet" contains a large amount of useful information to all who are engaged in the general practice of medicine. $5 a year. WM. C. HAZARD, agent, 52 John Street, New York.

The friends of public schools in New York City have reason for congratulation that Judge Hooper C. Van Vorst has been wisely appointed one of the Board of Commissioners by the Mayor of this city. He has been a steady and efficient friend of the public school system for many years, and is known as a scholar and high-toned Christian gentleman. Judge V. not long since dedicated "The Beatitudes," in verse, to his son,—a beautiful instance of the loving relationship between father and child.

Rev. John Lord, LL. D., whose admirable and instructive course of historical lectures have secured such large and appreciative audiences at noon, during the winter, has been invited to repeat at least some of them, by Bishop Potter, Robert L. Stuart, Dr. Doremus, and other of our most prominent citizens, at an hour more suitable for business men. He is certainly one of the most attractive of all our public lecturers; the ladies follow him in crowds.

"Fun the Best Physic; or, Everybody's Life-preserver," is a dollar-and-a-half book of 333 pages, containing twelve hundred of the Editor's "Precepts about Health," and everything. Said to be an excellent book to read on steamboats, railways, and horseback.

The Editor may be found at his office, 42 Broadway, New York, from ten to one o'clock, daily, and at those hours only. No consultations given at his residence at any time.

